# Genetic and Transmission Analysis of *Helicobacter pylori* Strains within a Family^1^

**DOI:** 10.3201/eid1010.040042

**Published:** 2004-10

**Authors:** Josette Raymond, Jean-Michel Thiberge, Catherine Chevalier, Nicolas Kalach, Michel Bergeret, Agnès Labigne, Catherine Dauga

**Affiliations:** * Hôpital Saint Vincent de Paul, Paris, France;; † Institut Pasteur, Paris, France;; ‡ Hôpital Saint Antoine, Lille, France

**Keywords:** Helicobacter pylori, family transmission, epidemiology, phylogeny, genetic diversity, research

## Abstract

To look for evidence of intrafamilial infection, we isolated 107 *Helicobacter pylori* clones from biopsied specimens taken from both parents and four children. We compared the sequences of two housekeeping genes (*hspA* and *glmM*) from these clones with those of 131 unrelated strains from patients living in different geographic regions. Strain relationships within the family were determined by analyzing allelic variation at both loci and building phylogenetic trees and by using multilocus sequence typing. Both *hspA*- and *glmM*-based phylogenetic trees showed East Asian and African branches. All samples from family members showed natural mixed infection. Identical alleles found in some strains isolated from the children and parents, but not in the strains isolated from unrelated patients, demonstrated that strains have circulated within the family. Several mechanisms, such as point mutations, intragenic recombination, and introduction of foreign (African) alleles, were shown to enhance strain diversity within the family.

## Abstract 2

Point mutations, intragenic recombination, and introduction of foreign alleles enhanced strain diversity within the family.

*Helicobacter pylori* is the major cause of chronic gastritis and peptic ulcers and must be treated to prevent relapse (*1*). *H. pylori* infection is considered a risk factor for developing gastric carcinoma (*2**,**3*). *H. pylori* strains appear to be spread by person-to-person contact (*4*), and DNA fingerprinting has provided evidence of transmission between family members (*5**–**9*). Clonal descent has been demonstrated by comparing alleles of genes such as *vacA*, *flaA*, and *flaB* of isolates infecting members of the same family (*10**–**12*) and by sequencing three housekeeping genes (*ureI*, *atpA*, and *ahpC*) (*13*). However, in all these studies, only one strain from each biopsied specimen was studied.

*H. pylori* is one of the most genetically diverse bacterial species, displaying from 2.7% to 8.0% of DNA sequence polymorphism (*14**–**16*). This diversity originates from both the clonal nature of the species and interstrain recombination events (*17**–**19*). Analysis of the sequences of housekeeping genes (*atpD*, *scoB*, *glnA* and *recA*) showed that strains cluster according to their geographic origins (*15**,**20*). Strains from the United States, Latin America, and Europe differ from those that are predominant in East Asia, coastal China, Hong Kong, Japan, south Asia (India), and Africa (*20**–**22*).

We estimated the allelic diversity of 20 isolates taken from two locations (fundus and antrum) in the stomach of each member of a family and studied person-to-person transmission within this family. To assess the genetic diversity and relationships between the isolates, we sequenced two housekeeping genes (*glmM* and *hspA*). These two genes are present in all *H. pylori* isolates and have been shown to be good tools to distinguish between isolates (*23**,**24*); g*lmM* sequences appear to be relatively well conserved between strains (*23*), whereas *hspA* sequences show enough variability to test geographic clustering (*24*).

## Materials and Methods

### Participants, Gastric Biopsy Samples, and Related *H. pylori* Isolates

The family consisted of two parents and four children; child 1, child 2, child 3, and child 4 were 14, 12, 8, and 2 years of age, respectively. The parents were from Algeria, and all children were born in France. The two parents had gastritis and the children had abdominal pain. Child 1 (index case-patient) was given appropriate triple therapy (lansoprazole plus amoxicillin plus clarithromycin for 7 days), according to the results of antimicrobial drug susceptibility testing. Despite treatment, this child remained infected with *H. pyloi*, which suggested treatment failure or reinfection. Informed consent was obtained from each adult and from the two eldest children. Parental consent was obtained for each child.

Biopsy samples were taken from the corpus and antrum of the stomach during endoscopic testing. Culture was performed as previously described (*23*). The Etest analysis (AB Biodisk, Solna, Sweden) showed that all isolates were susceptible to clarithromycin. When possible, 10 independent colonies were randomly selected from each primary culture and subcultured. A total of 107 independently subcultured isolates were stored as frozen suspensions. Repetitive sequence analysis previously found that freezing or subculturing strains had no effect on the stability of the *hspA* and *glmM* sequences.

### Unrelated *H. pylori* Isolates

Epidemiologically unrelated *H. pylori* isolates were collected from persons who underwent gastroduodenal endoscopy in various gastroenterology departments. Nineteen of the patients originally from Hong Kong (Queen Mary Hospital), 9 were from Senegal (C.H.U. Le Dantec, Dakar), 25 were from Venezuela (Facultad de Medicina, Universidad de los Andes, Merida), 22 were from Sweden (Karolinska Hospital, Stockholm), 18 from Iran (Pasteur Institute of Iran, Tehran), and 32 were from France (Saint Vincent de Paul Hospital, Paris). Strains Ovx 34, Takada 112/3 (isolated from a monkey), X47-2AL (isolated from a cat), 26695 (ATCC700392), J99 (ATCC700824) (16), 85P, and N6 (23) were used as reference strains.

### Molecular Techniques

Chromosomal DNA was extracted from 48-hour-old confluent cells by using the QIAamp Tissue Kit (Qiagen, Chatsworth, CA), according to the manufacturer's recommendations. A 487-bp segment containing the 384-bp *hspA* gene and a 294-bp fragment of the *glmM* gene was amplified by polymerase chain reaction (PCR) (*24**,**25*). Each purified PCR product was fully sequenced on both strands with an ABI310 automated DNA sequencer (Perkin-Elmer).

### Computer Analyses

Multiple DNA sequences were aligned with the CLUSTAL V program (*25*). Phylogenetic analyses were performed with the PAUP* software package, version 4.0 (*26*). Sequence distance matrices were established in pairwise comparisons by using the Kimura algorithm and a transition/transversion ratio of 3.88 and 4 for the *hspA* and *glmM* genes, respectively. Phylogenetic trees were constructed by the neighbor-joining method (*27*). Maximum-parsimony trees were obtained by 1,000 random addition heuristic search replicates without the branch-swapping option. A maximum-likelihood analysis was performed with the HKY85 model, which calculated the transition/transversion ratio and estimated the shape parameter of a γ distribution of rate variation among sites (*28*). Significance was evaluated by the jacknife method. Split decomposition was carried out with the Splits Tree program, version 2.4, (http://bibiserv.techfak.uni-bielefeld.de/splits/) with pairwise distance estimated with the Kimura model, to detect phylogenetic incongruence and show how recombination might affect the evolutionary relationships between *H. pylori* strains. Multilocus sequence typing (MLST) tools were used to delineate clusters of strains by using sequence output available from the MLST Web site (http://mlst.zoo.ox.ac.uk). Clusters of strains or clonal complexes were defined with the BURST algorithm on the MLST Web site. The sequences obtained during this study were assigned the following EMBL accession numbers: for *glmM* AJ809447–AJ809497 and for *hspA* AJ809893–AJ810031.

## Results

**Figure 1:**
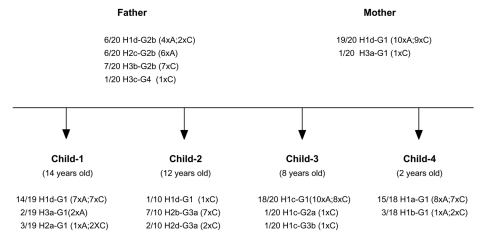
Number and genotypes of isolates from members of the family. The *hspA* and *glmM* alleles are designated by H and G, respectively. The alleles are numbered according to the cluster to which they belong on the phylogenetic trees (Figure 4). Small letters were assigned to the alleles that belong to the same cluster and differ by point mutations. (n x A; m x C), which shows the number (n or m) of colonies originating from the antrum (A) and the corpus (C), respectively.

We isolated and subcultured 107 *H. pylori* colonies from the antral and corpus biopsy samples collected from the family members (9 or 10 individual colonies per sample, except for child 2, for whom the antral biopsy culture was negative). We sequenced the *hspA* and *glmM* genes of all 107 colonies. The multiple alignments showed 11 different alleles for *hspA* (designated H1a,b,c,d; H2a,b,c,d; H3a,b,c) and six different alleles for *glmM* (designated G1; G2a,b; G3a,b; G4). Each strain was named by the combination of the *hspA* and the *glmM* alleles (e.g., H1d-G1 for an isolate harboring the H1d *hspA* allele and the G1 *glmM* allele, Figure 1). All family members had a natural mixed infection.

**Figure 2:**
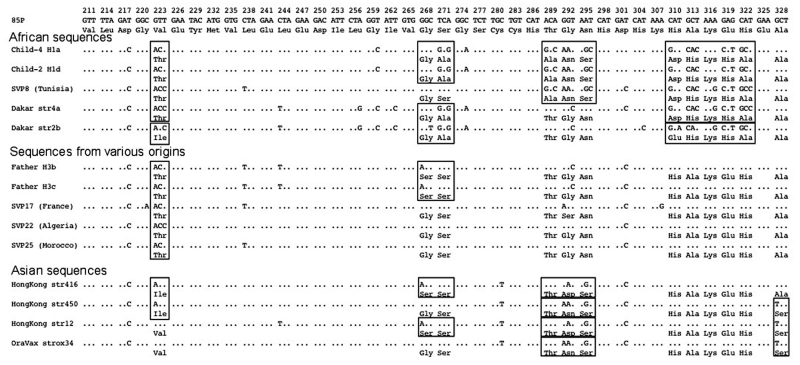
Mosaicism of the *hspA* 3´-end sequences.

**Figure 3:**
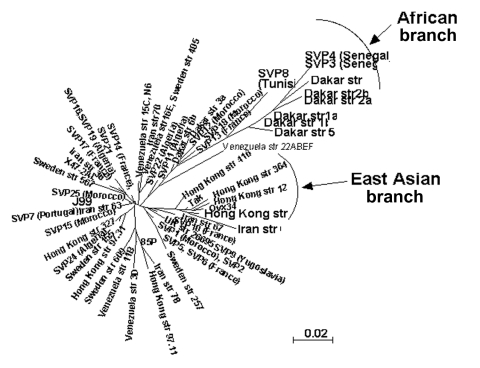
Neighbor-joining unrooted dendrogram for *hspA* sequences. The tree includes only 61 of the 131 *hspA* sequences representative of each cluster previously obtained from the global phylogenetic analysis. The scale bar indicates the number of substitutions per site according to the HKY index. Sequence names correspond to the geographic region of isolation followed by the strain number. SVP, Saint Vincent de Paul Hospital, Paris, France. The ethnic origin of French patients is mentioned in brackets when known.

We sequenced a 357-bp DNA region containing the entire *hspA* gene of 131 epidemiologically unrelated strains isolated from patients from different countries. The 19 Hong Kong strains contained a specific 9-bp signature, coding for [Thr-Asp-Ser] or [Thr-Asn-Ser] at positions 289–297 (*H. pylori* 85P, numbering system) of *hspA* (Figure 2). Sixteen of the Hong Kong strains belonged to the same branch of the *hspA*-based neighbor-joining tree (5 of these 16 Hong Kong strains are represented in Figure 3). Eight of the nine strains isolated in Dakar were grouped on the same branch as four strains from patients from Tunisia, Morocco, Algeria, and Senegal. This "African branch" was also visible on the parsimony consensus tree (data not shown). The African branch strains carried a specific 15-bp signature, coding for [Asp/Glu-His-Lys-His-Ala], at positions 310–324 of *hspA* (Figure 2). The other branches of the *hspA* phylogenetic trees consisted of isolates from patients of different ethnic origins.

**Figure 4:**
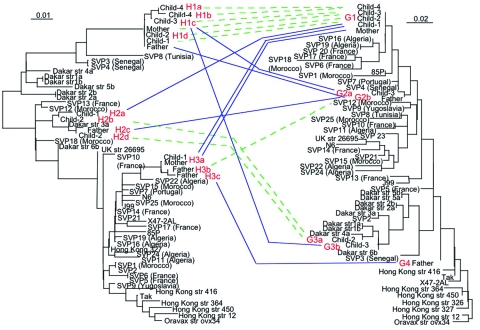
Relationships and genetic transfer hypotheses for the *hspA* and *glmM* alleles from *Helicobacter pylori* strains infecting members of the family. The phylogenetic trees based on *hspA* (left) and *glmM* (right) sequences were built by the maximum-likelihood method. The *hspA* sequences shown here originate from the same 47 strains from which the *glmM* sequence was determined. The names given to the sequences for family genes correspond to the name of the family member followed by the allele number. Dashed lines suggest coevolution of genes. Solid lines suggest genetic transfer.

We sequenced the *glmM* genes of 47 randomly selected isolates. The *glmM* phylogenetic trees based on the neighbor-joining (data not shown) and maximum likelihood methods (Figure 4) grouped most of the sequences from the Hong Kong strains in the same branch. Similarly, the *glmM* gene sequences of the strains from Senegal, Morocco, and Algeria formed an African branch, as did the *hspA* sequences.

Phylogenetic analyses of the *hspA* sequences of all the isolates (family plus different geographic regions) indicated three clusters (H1, H2, H3) (Figure 4). The H1a, H1b, H1c, and H1d allelic sequences belonged to the H1 cluster, which was present in all isolates from members of the family; these four alleles had nonsynonymous mutations at both ends of the gene. Cluster H2 included the H2a–H2d alleles, found in isolates from child 1, child 2, and the father; these alleles differed by six synonymous mutations (positions 20, 77, 89, 98, 131, and 188; 85P numbering system) and a nonsynonymous mutation at the 5´ end. Both the H1 and H2 clusters belonged to the African branch. Cluster H3 contained the three alleles H3a, H3b, and H3c, found in isolates from child 1, the father, and the mother; these alleles differed by a nonsynonymous mutation (5´ end) and a synonymous mutation (position 164). This third cluster was included in a heterogeneous group of strains isolated from humans of diverse ethnic origins.

Four clusters of *glmM* sequences (G1 to G4) were observed in the global phylogenetic tree (Figure 4). Allele G1 was strictly identical in strains isolated from the four children and the mother. The G2 cluster included the G2a sequence found in isolates from child 3 and the G2b sequence found in those from the father, differing by one synonymous mutation (position 21). Cluster G3, grouping the G3a allele from strains from child 2 and the G3b allele from those of child 3 that differed by one synonymous mutation (position 129), was included in the African branch. Finally, allele G4, represented by a single sequence from a father-derived strain, showed no close relationships with any of the other DNA sequences included in this study.

Several alleles of strains isolated from the parents and children belonged to the same clusters on the phylogenetic trees (Figure 4), and alleles within each cluster showed only isolated mutations. Comparison of the 3´ end sequences of the *hspA* gene showed a clear mosaic structure (Figure 2). A low shape parameter (0.42) of the γ distribution, which characterizes the heterogeneity of rate variation among sites of *hspA* sequences, was obtained. All of these facts led us to conclude that intragenic recombination events enhanced allelic diversity.

**Figure 5:**
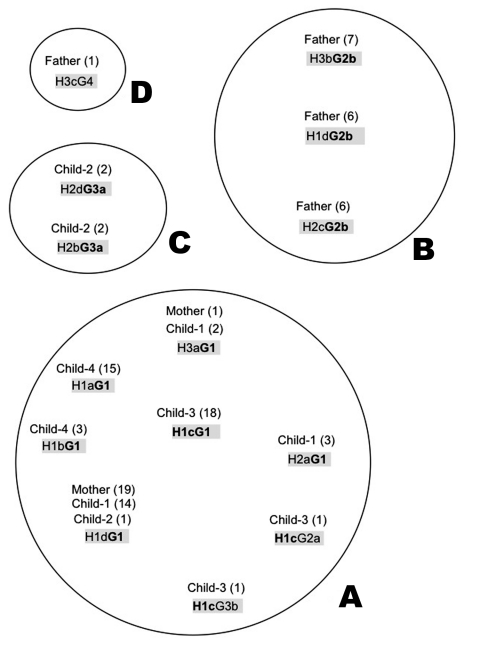
Multilocus sequence typing (MLST) analysis of family isolates. Clonal complexes were defined according to the alleles found associated with the highest number of different combinations among family strains. The names of clones are indicated by a letter: A, B, C or D. Alleles in bold are shared.

The diversity of allele associations allowed us to demonstrate that lateral gene transfer took place between strains within the family. For example, the H1c allele was associated with three different alleles (G1, G2a, and G3b) in the 20 strains from child 3 (Figure 1). In the same way, the G1 allele found in strains from the mother was associated with six *hspA* alleles (H1a, H1b, H1c, H1d, H2a, and H3a) in the strains isolated from the children. In these two examples, *hspA*- and *glmM*-associated alleles belonged to three genetically distant clusters. Thus, the two genes were acquired at different times even though they are located on the same chromosome (Figure 4). In addition, examination of phylogenetic trees of epidemiologically unrelated strains showed that some of the family alleles originated in Africa (H1a–H1d, H2a–H2d, G3a, and G3b) (Figure 3, Figure 4, Figure 5). The G3a and G3b alleles belonging to the African branch were only found in strains isolated from child 2 and child 3.

Analysis of strains from family members indicated four clonal complexes (A, B, C, D) (Figure 5). Strains harboring allele G1, found in six different combinations (H1aG1, H1bG1, H1cG1, H1dG1, H2aG1, H3aG1) or H1c, found in three different combinations (H1cG1, H1cG2a, H1cG3b), formed the main clonal subgroup, which included strains circulating between the mother and the children. The strains isolated from the father belonged to two subgroups (B and D). Subgroup D consisted of the strain H3cG4, containing a *glmM* allele of unknown origin, whereas subgroup B contained only the father-derived strains that harbored the G2b allele. The last subgroup (C) contained two strains isolated from child 2, characterized by two *hspA* and *glmM* African alleles (H2a, H2d, G3a).

## Discussion

Almost every *H. pylori* strain from the family and from patients living in different geographic regions had its own, unique DNA sequence (*15**,**22*). The level of synonymous divergence between the *hspA* and *glmM* alleles was similar to that observed for *vacA*, *flaA*, and *flaB*, whereas the level of nonsynonymous divergence for these two genes was slightly higher than the highest rate previously observed (2.9% versus 0.3%–2.5%) (*12*). This observation suggests that the two chosen DNA fragments (*hspA* and *glmM*) correspond to domains with a high level of mutations and encode proteins with few functional constraints.

By examining 10 colonies from each gastric biopsy sample from the antrum and fundus of each of the family members, we identified 11 different *hspA* alleles and six different *glmM* alleles (Figure 1). Phylogenetic analysis allowed us to map these different *hspA* and *glmM* alleles in three and four distinct branches of their respective trees, which suggested that they originated from different strains (Figure 4). The strain diversity seemed higher in the corpus than in the antrum (Figure 1). With the exception of the father, who was equally colonized (30%) by three of the four isolates, all family members appeared to be infected by a dominant strain (>70%). This multicolonization in a North African family is in agreement with data reporting that multicolonization is more frequent in countries in which *H. pylori* infection is highly prevalent (*29**,**30*).

The phylogenetic analyses based on the *hspA* and *glmM* sequences of strains from several regions of the world allowed us to build neighbor-joining and maximum likelihood trees. Several nodes were not defined on the parsimony consensus trees, which indicates many polytomies. This finding suggests a high level of homoplasy, resulting from a high level of parallel and convergent mutations that could be the result of recombination events (*15*). However, some of the clusters in these phylogenetic trees were reproduced with distance and maximum likelihood building algorithms and with the jacknife sampling method, which indicates the robustness of some of the nodes. Reproducible deep-branching clusters were associated with the geographic specificity of strains (Hong Kong or Africa). An East Asian branch has previously been reported for *hspA*, *glmM*, *vacA*, and other housekeeping genes (*15**,**22**,**31*) and for the *cag* pathogenicity island of *H. pylori* (*32*). We confirmed the clustering of East Asian strains and provided evidence in support of the recently proposed clustering of African strains (*22*).

Within the family, *hspA* and *glmM* alleles belonged to different clusters. Each of these individual groups contained two to four alleles that differed by a few mutations, consistent with genetic drift as previously described in *H. pylori* (*33*). Furthermore, genetic recombination events occurred between strains colonizing the family members (Figure 4). This study demonstrated that intragenic recombination events occurred during the evolution of the *hspA* and *glmM* genes, which resulted in mosaicism, networklike structures, or both (Figure 2), as has previously been reported for *vacA*, *flaA*, and some housekeeping genes (*12**,**34**,**35*). The discrepancies in the phylogenetic positions of a given strain based on the sequences of the *hspA* and *glmM* genes illustrate that lateral gene transfer has occurred among strains circulating within the family.

Some of the children were infected with strains harboring African alleles. We can speculate that foreign strains were introduced into the family through the children and added heterologous DNA that further increased the genetic diversity. However, we cannot exclude the possibility that, despite the large number of isolates from each family member, we undersampled the population and missed some alleles present in other isolates.

This study showed that all family members had mixed infections. Some of the alleles in the family strains were phylogenetically distinguishable, which allowed us to demonstrate conclusively the circulation of strains and the genetic exchange between family strains and confirming the intrafamilial dissemination of *H. pylori*. DNA fingerprinting methods have previously described heterogeneous populations of *H. pylori* clones derived from the culture of biopsies taken from a single patient (*13**,**15*). Randomly amplified polymorphic DNA analysis, PCR-restriction fragment length polymorphism, ribotyping, and pulsed-field gel electrophoresis have also demonstrated the clonal dissemination of strains within a family, concluding that family cross infection occurred or that the family members were infected by a common source (*5**,**8**,**13**,**20*). However, none of these studies involved sequencing genes from 20 individual colonies, which impeded the detection of minor variations between strains.

By sequencing the *hspA* and *glmM* genes of 20 isolates for each person from a family, we highlighted the involvement of some of the evolutionary mechanisms previously described in epidemiologically unrelated strains. This finding may clarify the mode of transmission and the evolution of the *H. pylori* genome.
